# Clinical Determinants and Prognosis of Left Ventricular Reverse Remodelling in Non-Ischemic Dilated Cardiomyopathy

**DOI:** 10.3390/jcdd9010020

**Published:** 2022-01-11

**Authors:** Carles Díez-López, Joel Salazar-Mendiguchía, Elena García-Romero, Lara Fuentes, Josep Lupón, Antoni Bayés-Genis, Nicolás Manito, Marta de Antonio, Pedro Moliner, Elisabet Zamora, Pablo Catalá-Ruiz, Miguel Caínzos-Achirica, Josep Comín-Colet, José González-Costello

**Affiliations:** 1 Advanced Heart Failure Unit, Department of Cardiology, Bellvitge University Hospital, L’Hospitalet de Llobregat, 08907 Barcelona, Spain; cdiez@bellvitgehospital.cat (C.D.-L.); jsalazarmg@gmail.com (J.S.-M.); e.garcia.r@bellvitgehospital.cat (E.G.-R.); nml@bellvitgehospital.cat (N.M.); pcatalaruiz@gmail.com (P.C.-R.); 2 Cardiology Department, Bellvitge University Hospital, L’Hospitalet de Llobregat, 08907 Barcelona, Spain; lara.fuentes@bellvitgehospital.cat (L.F.); jcomin@bellvitgehospital.cat (J.C.-C.); 3 Bellvitge Biomedical Research Institute (IDIBELL), L’Hospitalet de Llobregat, 08907 Barcelona, Spain; pmoliner@bellvitgehospital.cat; 4 Department of Medicine, University of Barcelona, 08907 Barcelona, Spain; 5 Heart Failure Unit, Department of Cardiology, Hospital Universitari Germans Trias i Pujol, 08916 Badalona, Spain; jlupon.germanstrias@gencat.cat (J.L.); abayesgenis@gmail.com (A.B.-G.); e.zamora@telefonica.net (E.Z.); 6 CIBERCV (CB16/11/00403), Instituto de Salud Carlos III, 28029 Madrid, Spain; mdeantonio@hotmail.com; 7 Community Heart Failure Program, Department of Cardiology, Bellvitge University Hospital, L’Hospitalet de Llobregat, 08907 Barcelona, Spain; 8 Johns Hopkins Ciccarone Center for the Prevention of Cardiovascular Disease, Johns Hopkins Medical Institutions, Baltimore, MD 21093, USA; miguel.cainzos@gmail.com; 9 RTI Health Solutions, Pharmacoepidemiology and Risk Management, 08028 Barcelona, Spain

**Keywords:** chronic heart failure, dilated cardiomyopathy, heart failure, left ventricular reverse remodelling, non-ischemic dilated cardiomyopathy

## Abstract

Aims: Non-ischaemic dilated cardiomyopathy (NIDCM) is characterized by left ventricular (LV) chamber enlargement and systolic dysfunction in the absence of coronary artery disease. Left ventricular reverse remodelling (LVRR) is the ability of a dilated ventricle to restore its normal size, shape and function. We sought to determine the frequency, clinical predictors and prognostic implications of LVRR, in a cohort of heart failure (HF) patients with NIDCM. Methods: We conducted a multicentre observational, retrospective cohort study of patients with NIDCM, with prospective serial echocardiography evaluations. LVRR was defined as an increase of ≥15% in left ventricular ejection fraction (LVEF) or as a LVEF increase ≥ 10% plus reduction of LV end-systolic diameter index ≥ 20%. We used multivariable logistic regression analyses to identify the baseline clinical predictors of LVRR and evaluate the prognostic impact of LVRR. Results: LVRR was achieved in 42.5% of 527 patients with NIDCM during the first year of follow-up (median LVEF 49%, median change +22%), Alcoholic aetiology, HF duration, baseline LVEF and the absence of LBBB (plus NT-proBNP levels when in the model), were the strongest predictors of LVRR. During a median follow-up of 47 months, 134 patients died (25.4%) and 7 patients (1.3%) received a heart transplant. Patients with LVRR presented better outcomes, regardless of other clinical conditions. Conclusions: In patients with NIDCM, LVRR was frequent and was associated with improved prognosis. Major clinical predictors of LVRR were alcoholic cardiomyopathy, absence of LBBB, shorter HF duration, and lower baseline LVEF and NT-proBNP levels. Our study advocates for clinical phenotyping of non-ischaemic dilated cardiomyopathy and intense gold-standard treatment optimization of patients according to current guidelines and recommendations in specialized HF units.

## 1. Introduction

Dilated cardiomyopathy (DCM) is defined as the presence of left ventricular or biventricular dilatation and systolic dysfunction that results in heart failure (HF), arrhythmias and thromboembolic events [1]. In spite of the latest advances in medical treatment, HF is related with high mortality rates and poor quality of life, and DCM is the leading cause of heart transplantation worldwide [2].

Although the DCM phenotype is extensive and pathophysiology complex, ischemic heart disease is the most prevailing cause of DCM [3]. From a practical point of view, non-ischaemic dilated cardiomyopathy (NIDCM) is a relatively common form of DCM, characterized by chamber enlargement and systolic dysfunction, in the absence of coronary artery disease [4,5]. NIDCM assembles a broad spectrum of aetiologies, which can be broadly classified as primary or genetic (i.e., without abnormal loading conditions causing systolic dysfunction), and secondary or non-genetic, a group composed by disorders such as hypertension, valvular heart disease, inflammatory myocardial disease (e.g., myocarditis), or the toxic effects of alcohol, drugs, or chemotherapy agents [4,5,6]. Additionally, the above-mentioned aetiologies may also occur in combination, as a part of the clinical picture.

The development of NIDCM involves a myriad of complex interactions within the cardiovascular system that are mostly mediated by neurohormonal factors, which promote a deleterious remodelling of the myocardium and lead to progressive cardiac chambers dilatation and clinical HF [7]. On the contrary, left ventricular reverse remodelling (LVRR) is defined as the ability of a dilated left ventricle to restore its normal size, shape and function, and it is associated with improved clinical outcomes in patients with DCM [8,9]. HF therapies such as angiotensin-converting enzyme inhibitors (ACEIs), angiotensin receptor blockers (ARBs), angiotensin receptor-neprilysin inhibitors (ARNIs), beta-adrenergic blocking agents (BB), mineralocorticoid receptor antagonists (MRAs) and cardiac resynchronization therapy (CRT), are capable of slowing and even reverting cardiac remodelling [10,11,12,13,14], and are associated with significant reductions in morbidity and mortality in HF with reduced ejection fraction [15,16,17,18]. In light of these data, LVRR has become a major therapeutic goal in patients with chronic HF (CHF), yet the clinical non-pharmacological predictors of LVRR are less well characterized, and the limited available evidence comes from studies that include a relatively small numbers of patients, from single centre cohorts, and yield inconsistent findings [19,20,21,22,23].

Therefore, the aims of the present study were: (1) To describe the frequency of LVRR in a well-characterized clinical cohort of patients with NIDCM followed in 2 specialized CHF units; (2) compare the characteristics of NIDCM patients with and without LVRR and identify clinical factors associated with LVRR; and (3) evaluate the prognostic implications, in terms of clinical events, of LVRR specifically in patients with NIDCM.

## 2. Methods

### 2.1. Study Design and Study Population

We conducted an observational, retrospective cohort study of CHF patients followed in two different specialized HF units from two different tertiary hospitals in Barcelona, Spain, between August 2004 and December 2016. For the present analysis, we included all patients from each of the two CHF units who had been diagnosed of NIDCM, had undergone prospective serial echocardiographic evaluations as part of their standard follow-up, and had been followed in the respective CHF unit for at least 1 year. For the purpose of the study, NIDCM was defined by a left ventricle ejection fraction (LVEF) < 45% in the absence of coronary artery disease. The later was ruled out either based on a negative result of a coronary angiography or, when not available, based on the simultaneous absence of angina, of a clinical suspicion of coronary artery disease, and on the presence of a negative result of a stress test.

The date of study entry for each patient was the date of start of follow-up in the respective CHF unit. Patients with a primary severe valvular disease considered to have a surgical indication were excluded from the study population.

### 2.2. Research Ethics

The study was approved by the local ethic committees of each study site and was conducted in accordance with the Declaration of Helsinki for research studies [24]. All participants provided written informed consent before study entry.

### 2.3. Baseline Evaluation

At inclusion in the respective CHF program (and therefore, at study entry), all patients underwent a detailed evaluation, including detailed medical history and physical examination, 12-lead baseline electrocardiogram, transthoracic echocardiography, and laboratory tests including levels of N-terminal prohormone of brain natriuretic peptide (NTproBNP) when they became available as part of a standard HF evaluation.

The CHF team that evaluated the patient at program/study entry determined whether the patient had NIDCM, as well as the most likely aetiology. Valvular etiology was defined as those with NIDCM in whom prior valvular disease has been already corrected and whose clinical symptoms were thought to be based on the underlying or persistent established myocardial disease. Alcoholic cardiomyopathy was defined as a NIDCM in whom the main etiology was thought to be a toxic effect of alcohol due to prior alcohol abuse.

### 2.4. 1-Year Assessment: Echocardiographic Evaluation, LVRR and Changes in Management

Patients were assessed with transthoracic echocardiography at CHF program baseline and after 1 year (±6 months) of CHF program enrolment. The left ventricular end-diastolic and end-systolic dimensions were measured using M-mode, and the LVEF was estimated using Simpson’s method. LVRR was defined as (a) left ventricle ejection fraction (LVEF) increase of ≥15% between the two exams, or as (b) LVEF increase ≥ 10% plus reduction of LV end-systolic diameter index ≥ 20% between the two exams. Changes in management at 1 year of follow-up compared to baseline were also evaluated.

### 2.5. Follow-Up and Event Ascertainment

As part of their follow-up in the respective CHF unit, patients were managed according to the CHF clinical practice guidelines of the European Society of Cardiology in place at each study period.

The primary study outcome was the occurrence of death or heart transplant (whichever happened first). Information on death events, heart transplantations and cause of death was obtained from the internal databases of each of the two CHF units, as well as from electronic medical record review and from direct contact with the patient (or their relatives if the patient had been lost to follow-up). The secondary study outcome was cardiovascular death or heart transplant.

### 2.6. Statistical Analyses

The baseline characteristics of the study participants were described overall and by LVRR status. Categorical variables were described using number and percentage, and continuous variables were described using median and interquartile range. Differences between patients with and without LVRR were tested using chi-squared tests for categorical variables (with Fisher’s correction when appropriate), and non-parametric tests for continuous variables.

The same approach was used to describe, and compare between LVRR groups, the baseline and 1-year echocardiographic characteristics of the study participants. We used multivariable logistic regression to identify baseline factors associated with LVRR. The NTproBNP value and time with heart failure were log transformed for the purpose of regression analysis as they followed a skewed distribution. First, we conducted univariate logistic regression analyses, with each of the potentially relevant factors associated with LVRR. All variables with a univariate association with LVRR *p* < 0.20 were then included in multivariable regression models. Because NTproBNP levels were not available in 33% of patients (i.e., patients included before 2006), we developed 2 models, one with NTproBNP and another without this parameter. Once all predictors had been included in the models, backwards stepwise selection was used to sequentially exclude non-statistically significant predictors with a *p* value > 0.10. We did this analysis in all patients, but also separately for patients with a LVEF < 35% and those with a LVEF ≥ 35%, for those with a history of HF of less or more than 3 months and for those with the follow-up echocardiogram performed less or more than 1 year after the initial echocardiogram.

To evaluate the prognostic impact of LVRR in each of the study outcomes, two approaches were used. First, we created Kaplan–Meier cumulative survival functions for individuals with and without LVRR starting after performing the second echo, and Log-Rank tests were used to compare them. Second, Cox Proportional Hazards regression was used to estimate the adjusted associations between LVRR, time to all-cause death, and time to cardiovascular death. For the latter analyses, the following hierarchical models were used: Model (1) unadjusted; Model (2) adjusted for age and sex; Model (3) adjusted for all of the predictors associated with LVRR in univariate analysis (*p* < 0.10) as well as predictors of all cause death or heart transplantation in univariate analysis (*p* < 0.10). Competing risk models were not used as death and heart transplant were grouped in a combined endpoint.

The threshold for statistical significance was a 2-tailed *p* value of 0.05. Analyses were conducted using SPSS Version 20 (SPSS, Inc., Chicago, IL, USA) and Stata software version 15 (StataCorp, College Station, TX, USA).

## 3. Results

### 3.1. Study Participants

The study population comprised a total of 527 patients with NIDCM and in whom at least two echocardiographic evaluations had been performed. Of them, 224 (42.5%) achieved LVRR in the first year of follow-up, and 303 (57.5%) did not.

### 3.2. Baseline Characteristics

The baseline characteristics of the study participants are presented in Table 1. Overall, median age was 60 years and 73% patients were males, and comorbidities were frequent and uniformly distributed along both of the study groups. Briefly, hypertension was reported in 54.5% of patients, diabetes mellitus in 32.6% and dyslipidemia in 43% of patients of the study population. Idiopathic DCM was the most frequent etiology, median HF duration at inclusion was 9 months, median LVEF was 27%, median NTproBNP levels were 1200 ng/L and 26% were in New York Heart Association (NYHA) functional class III-IV. The vast majority of patients were receiving betablocker and angiotensin-converting-enzyme inhibitor or angiotensin receptor blocker therapy (ACEI/ARB), up to 66% received aldosterone antagonists and CRT was implemented in 9.3% of patients of the overall population. There were no statistically significant differences between both groups regarding HF therapies and we did not find different prescription rates according to time of inclusion in the study (2004–2010 vs. 2010–2016).

Participants developing LVRR were younger and alcoholic etiology was more frequent than in non-LVRR patients. Individuals developing LVRR had shorter HF duration and better baseline NYHA functional class than their non-LVRR counterparts; they also had lower median NTproBNP levels at baseline, their QRS complex was narrower, and presence of left bundle branch block (LBBB) was less frequent.

### 3.3. Echocardiographic Assessment and Development of LVRR

The baseline and 1-year echocardiographic characteristics of the study participants are presented in Table 2. The follow-up echocardiogram was performed a median of 386 (IQR: 342–441) days after baseline. At baseline, median LVEF was slightly lower among individuals developing LVRR than among their non-LVRR counterparts. One year later, in patients with LVRR there was a marked improvement in all left ventricle parameters assessed, including an almost normal LVEF (median LVEF after 1 year 49%, median change +22%). Conversely, in those without LVRR, changes in left ventricle dimensions were close to null, median LVEF remaining almost unchanged (median LVEF after 1 year 31%, median change +3%).

### 3.4. Changes in Management at 1 Year of Follow-Up

Table 2 presents incident CRT implantation between the two echocardiographic assessments. Specifically, 5.2% of patients with no CRT at baseline were treated with CRT before the second echocardiography. Although there was a slightly higher use of CRT in the non-LVRR group, this difference was small and non-statistically significant.

### 3.5. Variables Associated with LVRR

Results of the logistic regression analyses identifying predictors of LVRR are presented in Table 3. In the univariate analyses, baseline age, hypertension, eGFR, NTproBNP, HF duration, NYHA functional class, indexed left ventricle diastolic diameter (iLVEDD), initial LVEF, absence of LBBB and QRS complex width were associated with the development of LVRR.

In the multivariable analyses, HF duration, initial LVEF and absence of LBBB (plus NTproBNP levels when included in the model) were the strongest independent clinical predictors of LVRR development in the subsequent year (Table 3). These predictors were slightly different for patients with LVEF above or below 35%, for those with a history of HF of less or more than 3 months and for those with the follow-up echocardiogram performed before or after 1 year. See Supplementary Tables S1–S6.

### 3.6. Follow-Up and Incident Events

Median follow-up was 47 (29–75) months. During this period there were 134 deaths, 76 of which were cardiovascular (14.4%) and 58 non-cardiovascular (11%). Moreover, 7 patients (1.3%) received a heart transplant.

### 3.7. Associations between LVRR and Clinical Outcomes

**Figure 1:**
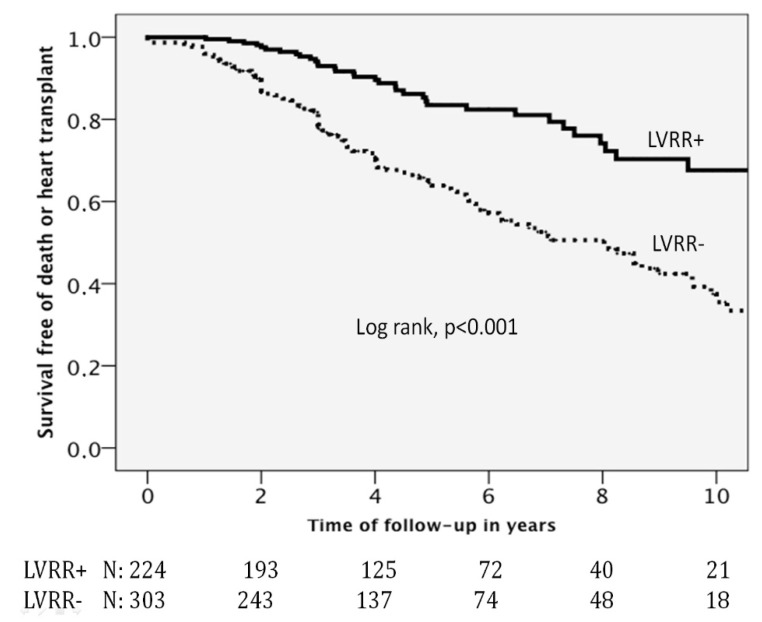
Kaplan–Meier survival function curves. Abbreviations: LVRR = left ventricular reverse remodelling. LVRR(+) = presence of left ventricular reverse remodelling; LVRR(−) = absence of left ventricular reverse remodelling.

Figure 1 displays the Kaplan–Meier survival function curves for the primary study outcome (all-cause death or heart transplant) and for the secondary outcome (cardiovascular death or heart transplant). Individuals who developed LVRR had a better event-free survival than those who did not (*p* value of the Log-Rank test < 0.001).

In the Cox regression analyses (Table 4), we confirmed a strong independent association between LVRR and a lower risk of the primary endpoint (HR 0.28, 0.13–0.57 95% CI, *p* < 0.001) and of the secondary endpoint (HR 0.12, 0.03–0.39 95% CI, *p* < 0.001). Likewise, the use of BB was also an independent predictor of survival free of death or heart transplantation (HR 0.32, 0.14–0.74 95%CI, *p*: 0.008), whereas baseline iLVEDD, NYHA functional class III-IV and older age were related with poorer outcomes.

## 4. Discussion

In a large cohort of patients with NIDC followed in two specialized HF units, we observed a high incidence of LVRR (43%) after 1 year of follow-up. In our study, besides etiology (patients with alcoholic cardiomyopathy developed LVRR more often), the strongest baseline clinical variables associated with subsequent LVRR were absence of LBBB, a shorter HF duration, a lower baseline LVEF and lower baseline levels of NTproBNP. LVRR was the strongest predictor of survival free from all-cause and cardiovascular death or heart transplant. To our knowledge, this is the largest study evaluating the incidence of LVRR in patients with NIDCM, and has important implications for understanding its real-life epidemiology, clinical determinants, prognosis, and implications for potential device and allocation.

### 4.1. Definition and Incidence of LVRR

The term ventricular remodelling refers to an alteration in ventricular architecture with associated increased volume and abnormal chamber configuration, driven at a histological level by a combination of pathological myocyte hypertrophy, myocyte apoptosis, myofibroblast proliferation and interstitial fibrosis [25]. This ventricular remodelling develops after myocardial injury and increased wall stress, and is associated with worsened outcomes; however, this adverse ventricular remodelling process can be reversed when the stress is removed or attenuated [26].

Other groups have studied the incidence of LVRR in patients with DCM, using different definitions. Brenyo et al. defined LVRR as a ≥15% reduction in left ventricular end-systolic volume at 1 year of follow-up. The cohort was based on the internal cardioverter defibrillator (ICD) arm of the MADIT-CRT, and thus included patients with ischemic aetiology and without pharmacological therapy. In those patients, the incidence of LVRR was 25% [27]. Merlo et al. also examined the incidence of LVRR, defined as an improvement in LVEF ≥ 10% and a decrease in LVEDD ≤ 10%, in patients with idiopathic DCM. The authors found a LVRR incidence of 37%, which was associated with a reduction in death or heart transplant during follow-up [28]. They also evaluated the incidence of persistent recovery of normal LVEF and dimension, defining persistent healing as a LVEF ≥ 50% and an indexed left ventricular end-diastolic diameter (iLVEDD) ≤ 33 mm/m^2^. After 19 months, 15% patients were apparently healed, observing persistent apparent healing in 9% of patients at long-term follow-up (103 months) [29]. Finally, other groups have defined LVRR as a LVEF > 35%, finding an incidence of LVRR of 46% [27], although this cohort included a mixed population of ischaemic and non-ischaemic patients with DCM.

The incidence of LVRR in our population was slightly higher to that reported in other studies. This may be explained by several factors: (1) the exclusion of ischaemic DCM; (2) the inclusion of aetiologies such as alcoholic or hypertensive cardiomyopathy, a subgroup of patients who can normalize ventricular function with medical therapy and toxic consumption cessation; (3) the relatively short median duration of CHF at study entry; and (4) the fact that participants were followed in multidisciplinary HF units, which may have resulted in better up-titration of neurohormonal pharmacotherapies than in other cohorts. The incidence of LVRR also depends on the definition used of reverse remodelling, nevertheless, this would unlikely explain the incidence observed in our study, provided that the criteria used were very stringent.

### 4.2. Variables Associated with LVRR

Based on our results, the ECG appears as a powerful prognostic tool in patients with NIDCM. Specifically, absence of LBBB, which is a marker of less advanced disease, was strongly associated with LVRR development. LBBB has been previously described as a powerful predictor of worse outcomes in patients with DCM [23,24,25,26,27,28,30]. This finding is consistent with the observations by Merlo et al. [28] and Lupón et al. [31], although the latter included also patients with ischemic etiology. Importantly, although less than half of patients with LBBB were treated with CRT, it is important to note that this proportion was assessed only one year after cohort entry.

A lower baseline LVEF was also associated with subsequent LVRR development, in our study and also in the studies by Arad et al. [22] and Lupón et al. [31]. From a numerical point of view, it must be noted that is more likely to find LVRR when the baseline LVEF is lower, as the room for improvement is greater. Furthermore, this association may reflect recent disease or sub-acute presentations, in which earlier intervention could yield a better chance of improvement. Of note, LVEF at baseline remained associated with LVRR even in those patients with a LVEF ≥ 35%.

The same reasoning applies to duration of CHF at study entry, which was also associated with LVRR (the shorter the duration, the higher the odds of LVRR). Previous studies designed to find predictors of reverse remodelling focused mainly on patients with recent-onset cardiomyopathy, including patients with a history of cardiac symptoms shorter than 6 months [20,32]. In our study, the median heart failure duration was of 9 months, and shorter HF duration came out as an independent predictor of LVRR in our population. Of note, the presence of LVRR has been shown to be a favorable prognostic indicator irrespective of its timing (early vs. delayed LVRR) [33].

In patients in whom we had NTproBNP values available, this variable was also associated with LVRR, possibly reflecting less advanced NICDM and therefore, it suggests that it could be more feasible to get LVRR after optimized medical therapy. Moreover, a past medical history of hypertension was also a predictor of LVRR.

Subgroup analysis showed that these predictors of LVRR were similar for patients with lower or higher LVEF. We also found that in patients with a history of HF of more than 3 months these predictors remained quite similar to those of the global cohort, providing unique information on the effect of a specialized HF clinic in achieving LVRR in patients who have chronic HF.

### 4.3. Prognosis of LVRR and Clinical Implications

Predicting prognosis in patients with NIDCM is challenging, yet our results have direct implications for the management of these patients. Based on our results, clinicians should always warrant a period of watchful waiting regarding ICD implantation, since the development of LVRR could be expected in almost half of the patients and it is associated with lower rates of death and heart transplantation. Although ICD implantation is a live-saving therapy [1], its implantation carries a significant risk of complications both in the surgical initial process and during follow up [34]. This is particularly important, considering the findings of the DANISH Registry, in which prophylactic ICD implantation in patients with NIDCM was not associated with improved survival [35]. For all of the above, clinical guidelines emphasize that devices should only be implanted once the patient is on optimal medical therapy, including up-titration of neurohormonal medication to the maximally tolerated doses [1]. Given the low cardiovascular mortality in patients who achieved LVRR, our study provides simple clinical tools to evaluate patients with lower probability of achieving LVRR, and therefore these patients may undergo an early deeper phenotyping using additional clinical tools such as cardiac magnetic resonance or even genetic testing when indicated, to early evaluate for ICD as primary prevention and advanced therapies such as heart transplant and mechanical circulatory support in specific advanced heart failure units.

### 4.4. Study Strengths

Our study has several strengths. To our knowledge, to date this is one of the largest studies examining the incidence and predictors of LVRR, overcoming some of the limitations of prior, smaller cohorts. Moreover, inclusion of patients from two centres with comprehensive HF profiles increased the generalizability of the findings. In addition, the fact that patients were followed in high-quality, multidisciplinary CHF units allowed to characterize the incidence and prognostic implications of LVRR in the setting of optimized CHF management.

### 4.5. Study Limitations

Our study has also some limitations that are worth mentioning. First, we restricted inclusion to patients with at least one follow-up echocardiogram, performed 1 year after study entry. This may have resulted in a depletion of the most severe cases, increasing the incidence of LVRR. Nevertheless, this limitation is almost impossible to overcome in studies assessing 1-year changes in echocardiographic parameters.

Second, echocardiographic variables such as diastolic function, strain rate or pulmonary hypertension could not be incorporated to the definition of LVRR due to the lack of availability of this data. Nevertheless, our parsimonious definition is similar to that used in previous studies and can be easily implemented in most echocardiographic labs. Similarly, no data was available regarding late gadolinium enhancement, which is considered a predictor of ventricular arrhythmias or left ventricular reverse remodelling respectively [36].

Third, information on drug doses was not available either, neither at baseline nor during follow-up. This precluded assessing differences in the attainment of target doses for key pharmacotherapies such as betablockers and ACEI/ARBs, between patients who did/did not develop LVRR. Nevertheless, use of ICD and CRT was similar in both groups both at baseline and at 1 year, making large differences between groups in their pharmacological management very unlikely. Furthermore, because the present study was done before the PARADIGM-HF study, patients did not have access to ARNIs, which could have resulted in a higher number of patients experiencing LVRR [37].

Fourth, in the time of our present study, the use of genetic testing and deeper phenotypic clustering to improve characterization of NIDCM patients was uncommon and thus, there was no opportunity to enhance treatment specificity. It is of note that, in recent years, the importance of deeper characterization including genetic, transcriptomic, anatomopathological and autoimmune analyses in NIDCM has been greatly emphasized to tailor the medical treatment, improve the prognostic estimations and perform adequate genetic counselling [3,4,5,38].

Fifth, in our study, one of the main factors that correlate with LVRR is HF duration. We nonetheless recognize that those patients with longer HF duration might have been previously under medical treatment and might have less room for treatment effect which could constitute a potential bias. However, this limitation is present in any previous observational studies were HF duration has been recognized for a long time as one of the main factors that correlate with LVRR, meaning that patients should be referred early to specialized HF units to optimize treatment effect.

Finally, the levels of NTproBNP were not available in 33% of study participants. Likewise, levels of other biomarkers were not available either. In one study, NTproBNP was not an independent predictor of LVRR and the only biomarker that predicted LVRR was ST2 (a marker of fibrosis) [39].

## 5. Conclusions

In a large cohort of CHF patients with NIDCM under optimal medical management from two specialized CHF units, LVRR was frequent after 1 year of follow-up, and was associated with improved prognosis. Besides etiology (patients with alcoholic cardiomyopathy developed LVRR more often), the strongest baseline clinical variables associated with reverse remodelling were the absence of LBBB, shorter HF duration, lower baseline LVEF, and lower baseline levels of NT-proBNP. Our findings advocate for a deeper phenotyping of NIDC, underscore the usefulness of widely available clinical parameters to improve the management of CHF and to perseverate on the need for intense treatment optimization according to current guidelines and recommendations in specialized HF units.

## Figures and Tables

**Table 1:** Baseline characteristics of the study participants, overall and by LVRR.

	Overall N = 527	LVRR N = 224	No LVRR N = 303	*p* Value
***Demographics***				
Age (years)	60 (51–71)	58 (49–68)	64 (54–72)	<0.001
Male sex	385 (73.1)	161 (71.9)	224 (73.9)	0.60
***Risk factors and comorbidities***				
Hypertension	287 (54.5)	133 (59.4)	154 (50.8)	0.051
Diabetes mellitus	172 (32.6)	69 (30.8)	103 (34.0)	0.440
Dyslipidemia	227 (43.0)	92 (41.1)	135 (44.6)	0.425
COPD	89 (16.9)	43 (19.2)	46 (15.2)	0.224
***Hemodynamics***				
SBP (mmHg)	120 (110–136)	120 (108–140)	120 (110–135)	0.486
Heart rate (bpm)	72 (64–80)	73 (63–80)	72 (65–80)	0.835
***Blood tests***				
Haemoglobin (g/L)	13.6 (12.3–14.7)	13.7 (12.4–14.8)	13.6 (12.3–14.7)	0.319
eGFR (mL/min)	67.8 (48–94.7)	74.5 (52–97.7)	64.7 (46–90)	0.034
NT-proBNP (ng/L)	1201 (434–2937)	840 (341–2207)	1446 (543–3476)	0.001
***HF aetiology***				
IDC	206 (39.1)	82 (36.6)	124 (40.9)	0.37
Hypertensive	65 (12.3)	27 (12.1)	38 (12.5)	0.87
Alcoholic	69 (13.1)	45 (20.1)	24 (7.9)	<0.001
Drug-induced	23 (4.4)	12 (5.4)	11 (3.6)	0.34
Valvular	72 (13.7)	21 (9.4)	51 (16.8)	0.01
Other ^a^	92 (17.5)	37 (16.5)	55 (18.2)	0.63
***HF characteristics***				
HF duration (months)	9 (2–52)	3 (1–19)	21 (3–69)	<0.001
III-IV NYHA class	137 (26.0)	47 (21.0)	90 (30.0)	0.022
Initial iLVEDD (mm)	35.2 (32–38)	34.8 (32–42)	35.6 (32–40)	0.124
Initial LVEF (%)	27 (22–32)	25 (20–30)	28 (23–33)	<0.001
***Electrocardiogram***				
Sinus rhythm	385 (73.1)	173 (77.2)	212 (70.0)	0.063
Absence of LBBB	408 (77.4)	191 (85.2)	217 (71.6)	<0.001
QRS complex (ms)	120 (80–145)	100 (80–120)	120 (90–160)	<0.001
***Devices***				
ICD	64 (12.0)	23 (10.0)	41 (13.0)	0.10
CRT	49 (9.3)	19 (8.5)	30 (9.9)	0.738
***Pharmacotherapies***				
ACEI/ARB	505 (95.8)	215 (96.0)	290 (95.7)	0.877
Betablocker	484 (91.8)	211 (94.0)	273 (90.0)	0.089
Aldosterone antagonist	350 (66.4)	145 (64.7)	205 (67.7)	0.482
Diuretics	478 (90.7)	207 (92.4)	271 (89.4)	0.246

Data presented as number (%) for categorical variables, and as median (interquartile range) for continuous variables. ^a^ Including non-compaction cardiomyopathy, tachycardiomyopathy, and other causes. Abbreviations: ACEI/ARB: angiotensin converting enzyme inhibitors/angiotensin II receptor antagonists; COPD = chronic obstructive pulmonary disease; CRT = cardiac resynchronization therapy; eGFR = estimated glomerular filtration rate; HF = heart failure; ICD = implantable cardioverter-defibrillator; IDC = idiopathic dilated cardiomyopathy; iLVEDD = indexed left ventricular end-diastolic diameter; LBBB = left bundle branch block; LVEF = left ventricle ejection fraction; LVRR = left ventricular reverse remodelling; NTproBNP = N-terminal prohormone of brain natriuretic peptide; NYHA = New York Heart Association; SBP = systolic blood pressure.

**Table 2:** Baseline and 1-year echocardiographic characteristics of the study population and changes in medical, overall and by LVRR.

	Overall N = 527	LVRR N = 224	No LVRR N = 303	*p* Value
***Baseline parameters***				
LVEF (%)	27 (22–32)	25 (20–30)	28 (23–33)	<0.001
iLVEDD (mm/m^2^)	35.2 (32–38)	34.8 (31.6–42)	35.6 (31.7–40.3)	0.12
iLVESD (mm/m^2^)	29.4 (25.5–33.2)	29.5 (26–33)	29.3 (25.4–33.5)	0.99
***1-year parameters***				
LVEF (%)	39 (29–48)	49 (43–55)	31 (25–37)	<0.001
iLVEDD (mm/m^2^)	32.7 (29.4–36.8)	30 (28–33)	35 (31.4–39.3)	<0.001
iLVESD(mm/m^2^)	25 (21.3–29.7)	21.7 (18.8–24.8)	28.3 (24.3–32.7)	<0.001
***Median change***				
LVEF (%)	9 (2–21)	22 (18–29)	3 (0–7)	<0.001
iLVEDD (%)	−5.6 (−13.8, −1.6)	−13 (−19, −6.8)	0 (−6.6, 4.1)	<0.001
iLVESD (%)	−11 (−23.5, 0)	−24.5 (−43, −16)	−1.9 (−11, 5.6)	<0.001
**Changes in management between assessments**				
Implantation of CRT ^a^	26 (5.2)	10 (4.7)	16 (5.5)	0.197

Data presented as number (%) for categorical variables, and as median (interquartile range) for continuous variables. ^a^ The denominators for these calculations were the number of patients with no CRT at baseline (N = 504). Abbreviations: CRT = cardiac resynchronization therapy; iLVEDD = indexed left ventricular end-diastolic diameter; iLVESD = indexed left ventricular end-systolic diameter; LVEF = left ventricle ejection fraction; LVRR = left ventricular reverse remodelling.

**Table 3:** Analysis of potential variables associated with LVRR.

	Univariate Analysis			Multivariable Analysis, NTProBNP Included			Multivariable Analysis, NTProBNP Not Included		
	OR	95% CI	*p* Value	OR	95% CI	*p* Value	OR	95% CI	*p* Value
Age (years)	0.98	0.96–0.99	<0.001	0.98	0.96–0.99	0.045	0.98	0.97–0.99	0.038
Male sex	1.10	0.75–1.6	0.60	–	–	–	–	–	–
Hypertension	1.41	1–2.00	0.05	–	–	–	1.59	1.07–2.37	0.022
Diabetes Mellitus	0.86	0.60–1.25	0.44	–	–	–	–	–	–
Dyslipemia	0.87	0.61–1.23	0.42	–	–	–	–	–	–
COPD	1.32	0.84–2.10	0.22	–	–	–	–	–	–
SBP (mmHg)	1.01	1.00–1.01	0.28	–	–	–	–	–	–
Heart rate (bpm)	1.00	0.98–1.01	0.26	–	–	–	–	–	–
Haemoglobin (g/L)	1.05	0.95–1.15	0.36	–	–	–	–	–	–
eGFR(mL/min)	1.00	0.99–1.01	0.098	0.99	0.98–0.99	0.002	–	–	–
logNT-proBNP (ng/L)	0.78	0.67–0.92	0.002	0.66	0.53–0.81	<0.001	–	–	–
logHF duration (months)	0.72	0.65–0.80	<0.001	0.78	0.68–0.90	0.001	0.73	0.66–0.82	<0.001
III-IV NYHA class	0.62	0.41–0.93	0.02	–	–	–	–	–	–
Initial iLVEDD (mm)	0.95	0.92–0.99	0.04	0.95	0.90–0.99	0.046	–	–	–
Initial LVEF (%)	0.95	0.93–0.97	<0.001	0.90	0.86–0.94	<0.001	0.93	0.90–0.95	<0.001
Sinus rhythm	1.45	0.98–2.1	0.64	–	–	–	–	–	–
Absence of LBBB	2.3	1.47–3.60	<0.001	2.94	1.61–5.36	<0.001	2.42	1.47–3.97	0.001
QRS complex (ms)	0.99	0.98–0.99	<0.001	–	–	–	–	–	–
ICD	0.73	0.42–1.26	0.26	–	–	–	–	–	–
CRT	0.84	0.46–1.54	0.60	–	–	–	–	–	–
ACEI/ARB	1.00	0.45–2.60	0.90	–	–	–	–	–	–
Betablocker	1.71	0.90–3.50	0.09	–	–	–	–	–	–
Aldosterone antagonist	0.88	0.60–1.26	0.50	–	–	–	–	–	–
Diuretics	1.4	0.78–2.66	0.25	–	–	–	–	–	–

Data presented as odds ratios and 95% confidence intervals from the logistic regression models. Abbreviations: ACEI/ARB: angiotensin converting enzyme inhibitors/angiotensin II receptor antagonists; COPD = chronic obstructive pulmonary disease; CRT = cardiac resynchronization therapy; eGFR = estimated glomerular filtration rate; HF = heart failure; ICD = implantable cardioverter-defibrillator; iLVEDD = indexed left ventricular end-diastolic diameter; LBBB = left bundle branch block; LVEF = left ventricle ejection fraction; LVRR = left ventricular reverse remodelling; NTproBNP = N-terminal prohormone of brain natriuretic peptide; NYHA = New York Heart Association; SBP = systolic blood pressure.

**Table 4:** Associations between LVRR and the primary study endpoint (all-cause death or heart transplantation).

	Model 1			Model 2			Model 3		
	HR	95% CI	*p* Value	HR	95% CI	*p* Value	HR	95% CI	*p* Value
LVRR	0.35	0.24–0.51	<0.001	0.40	0.27–0.58	<0.001	0.29	0.14–0.62	0.001
Age (years)	–	–	–	1.05	1.03–1.06	<0.001	1.03	0.99–1.07	0.0765
Male sex	–	–	–	1.12	0.78–1.60	0.541	0.65	0.33–1.30	0.656
Hypertension	–	–	–	–	–	–	0.87	0.48–1.59	0.660
Diabetes Mellitus	–	–	–	–	–	–	1.99	1.09–3.64	0.025
eGFR(mL/min)	–	–	–	–	–	–	0.99	0.98–1.01	0.274
Hemoglobin (g/L)	–	–	–	–	–	–	1.11	0.92–1.33	0.277
logNT-proBNP (ng/L)	–	–	–	–	–	–	1.21	0.88–1.67	0.239
logHF duration (months)	–	–	–	–	–	–	1.07	0.89–1.27	0.466
III-IV NYHA class	–	–	–	–	–	–	1.95	1.04–3.65	0.037
Initial iLVEDD (mm)	–	–	–	–	–	–	0.93	0.87–0.99	0.037
Initial LVEF (%)	–	–	–	–	–	–	0.99	0.94–1.05	0.696
Sinus ryhthm	–	–	–	–	–	–	1.06	0.54–2.07	0.875
Absence of LBBB	–	–	–	–	–	–	1.24	0.55–2.89	0.602
QRS complex (ms)	–	–	–	–	–	–	1.00	0.99–1.01	0.959
Aldosterone Antagonist	–	–	–	–	–	–	1.92	0.88–4.16	0.098
Diuretic	–	–	–	–	–	–	0.63	0.18–2.27	0.486
Statin	–	–	–	–	–	–	0.68	0.37–1.25	0.226
Betablocker	–	–	–	–	–	–	0.40	0.16–0.99	0.037

Results presented as HRs and 95% CIs from the Cox Proportional Hazards regression models: Model 1 was unadjusted; Model 2 adjusted for age and sex; Model 3 further adjusted for the variables that were found to be associated with LVRR in the logistic regression analyses Abbreviations: CI = confidence interval; eGFR = estimated glomerular filtration rate; HF = heart failure; HR = hazard ratio; iLVEDD = indexed left ventricular end-diastolic diameter; LBBB = left bundle branch block; LVEF = left ventricle ejection fraction; LVRR = left ventricular reverse remodelling; NTproBNP = N-terminal prohormone of brain natriuretic peptide; NYHA = New York Heart Association.

## Data Availability

No available.

## Supplementary Materials

The following are available online at https://www.mdpi.com/article/10.3390/jcdd9010020/s1, Table S1: Analysis of potential predictors of LVRR in patients with LVEF < 35% (N = 433); Table S2. Analysis of potential predictors of LVRR in patients with LVEF ≥ 35% (N = 94); Table S3. Analysis of potential predictors of LVRR in patients with a history of HF duration of ≤3 months (N = 190); Table S4. Analysis of potential predictors of LVRR in patients with a history of HF duration of >3 months (N = 337); Table S5. Analysis of potential predictors of LVRR in patients with the follow-up echocardiogram performed 1 year or less after the initial echocardiogram (N = 180); Table S6. Analysis of potential predictors of LVRR in patients with the follow-up echocardiogram performed 1 year after the initial echocardiogram (N = 347).

## Abbreviations

ACEI	angiotensin converting enzyme inhibitor
ARB	angiotensin II receptor antagonist
ARNI	angiotensin receptor-neprilysin inhibitor
BB	beta-blockers
CHF	chronic heart failure
DCM	dilated cardiomyopathy
COPD	chronic obstructive pulmonary disease
CRT	cardiac resynchronization therapy
eGFR	estimated glomerular filtration rate
HF	heart failure
ICD	implantable cardioverter-defibrillator
IDC	idiopathic dilated cardiomyopathy
iLVEDD	indexed left ventricular end-diastolic diameter
iLVESD	indexed left ventricular end-systolic diameter
LBBB	left bundle branch block
LV	left ventricle
LVEF	left ventricle ejection fraction
LVRR	left ventricular reverse remodelling
MRA	mineralocorticoid receptor antagonists
NIDCM	non-ischaemic dilated cardiomyopathy
NTproBNP	N-terminal prohormone of brain natriuretic peptide
NYHA	New York Heart Association
SBP	systolic blood pressure
