# Supplementary material for: Clinical Determinants and Prognosis of Left Ventricular Reverse Remodelling in Non-Ischemic Dilated Cardiomyopathy
Source: J Cardiovasc Dev Dis. 2022 Jan 11;9(1):20. doi: 10.3390/jcdd9010020 (PMC8778173; doi:10.3390/jcdd9010020)
Supplement: Supplementary file 1 [file jcdd-09-00020-s001.zip › jcdd-1476998-supplementary/Supplemental TableS1.pdf]

Table S1. Analysis of potential predictors of LVRR in patients with LVEF < 35% (N=433).

**Variable**	**Multivariate analysis, NTproBNP included**			**Multivariate analysis, NTproBNP not included**		
	**OR**	**95% CI**	**P value**	**OR**	**95% CI**	**P value**
Age	0.97	0.95-1.00	0.060	0.98	0.97-1.00	0.101
Hypertension				0.65	0.42-1.01	0.054
eGFR (ml/min)	0.98	0.97-0.99	0.012			
logNT-proBNP (ng/L)	0.65	0.51-0.82.	<0.001			
logHF duration (months)	0.81	0.69-0.94	0.006	0.77	0.68-0.87	<0.001
Initial iLVEDD (mm)	0.94	0.89-0.99	0.040	0.95	0.91-0.99	0.032
Initial LVEF (%)	0.91	0.86-0.96.	0.001	0.93	0.89-0.97	<0.001
QRS complex (ms)				0.99	0.98-0.99	0.005
Absence of LBBB	2.37	1.27-4.44	0.007			

Data presented as odds ratios and 95% confidence intervals from the logistic regression models. Abbreviations: eGFR = estimated glomerular filtration rate; HF = heart failure; iLVEDD = indexed left ventricular end-diastolic diameter; LBBB = left bundle branch block; LVEF = left ventricle ejection fraction; LVRR = left ventricular reverse remodeling; NTproBNP = N-terminal prohormone of brain natriuretic peptide.
