# Supplementary material for: Clinical Determinants and Prognosis of Left Ventricular Reverse Remodelling in Non-Ischemic Dilated Cardiomyopathy
Source: J Cardiovasc Dev Dis. 2022 Jan 11;9(1):20. doi: 10.3390/jcdd9010020 (PMC8778173; doi:10.3390/jcdd9010020)
Supplement: Supplementary file 1 [file jcdd-09-00020-s001.zip › jcdd-1476998-supplementary/Supplemental TableS2.pdf]

Table S2. Analysis of potential predictors of LVRR in patients with LVEF ≥ 35% (N=94).

Variable	Multivariate analysis, NTproBNP included			Multivariate analysis, NTproBNP not included		
	**OR**	**95% CI**	**P value**	**OR**	**95% CI**	**P value**
Age				0.96	0.92-1.00	0.094
eGFR (ml/min)	0.97	0.95-1.00	0.067			
logNT-proBNP (ng/L)	0.53	0.27-1.02	0.058			
logHF duration (months)	0.58	0.35-0.96	0.034	0.62	0.43-0.88	0.009
Initial LVEF (%)	0.71	0.51-0.98	0.041	0.61	0.43-0.87	0.007

Data presented as odds ratios and 95% confidence intervals from the logistic regression models. Abbreviations: eGFR = estimated glomerular filtration rate; HF = heart failure; LVEF = left ventricle ejection fraction; LVRR = left ventricular reverse remodeling; NTproBNP = N-terminal prohormone of brain natriuretic peptide.
