# Supplementary material for: Clinical Determinants and Prognosis of Left Ventricular Reverse Remodelling in Non-Ischemic Dilated Cardiomyopathy
Source: J Cardiovasc Dev Dis. 2022 Jan 11;9(1):20. doi: 10.3390/jcdd9010020 (PMC8778173; doi:10.3390/jcdd9010020)
Supplement: Supplementary file 1 [file jcdd-09-00020-s001.zip › jcdd-1476998-supplementary/Supplemental TableS3.pdf]

Table S3. Analysis of potential predictors of LVRR in patients with a history of HF duration of ≤ 3 months (N=190).

**Variable**	**Multivariate analysis, NTproBNP included**			**Multivariate analysis, NTproBNP not included**		
	**OR**	**95% CI**	**P value**	**OR**	**95% CI**	**P value**
Age				0.97	0.94-0.99	0.008
Hypertension				1.82	0.93-3.59	0.082
logNT-proBNP (ng/L)	0.77	0.58-1.02	0.071			
Initial LVEF (%)	0.91	0.84-0.98	0.019	0.95	0.90-1.00	0.072
QRS complex (ms)	0.98	0.96-0.99	<0.001	0.98	0.97-0.99	0.002

Data presented as odds ratios and 95% confidence intervals from the logistic regression models. Abbreviations: LVEF = left ventricle ejection fraction; LVRR = left ventricular reverse remodeling; NTproBNP = N-terminal prohormone of brain natriuretic peptide.
