# Supplementary material for: Clinical Determinants and Prognosis of Left Ventricular Reverse Remodelling in Non-Ischemic Dilated Cardiomyopathy
Source: J Cardiovasc Dev Dis. 2022 Jan 11;9(1):20. doi: 10.3390/jcdd9010020 (PMC8778173; doi:10.3390/jcdd9010020)
Supplement: Supplementary file 1 [file jcdd-09-00020-s001.zip › jcdd-1476998-supplementary/Supplemental TableS4.pdf]

Table S4. Analysis of potential predictors of LVRR in patients with a history of HF duration of > 3 months (N=337).

**Variable**	**Multivariate analysis, NTproBNP included**			**Multivariate analysis, NTproBNP not included**		
	**OR**	**95% CI**	**P value**	**OR**	**95% CI**	**P value**
Hypertension				1.83	1.1-3.06	0.020
eGFR (ml/min)	0.98	0.97-0.99	0.015			
logNT-proBNP (ng/L)	0.61	0.46-0.82	0.001			
logHF duration (months)	0.69	0.54-0.88	0.003	0.68	0.55-0.83	<0.001
Initial LVEF (%)	0.91	0.87-0.95	<0.001	0.92	0.89-0.96	<0.001
Absence of LBBB	2.68	1.31-5.49	0.007	2.22	1.22-4.03	0.009

Data presented as odds ratios and 95% confidence intervals from the logistic regression models. Abbreviations: eGFR = estimated glomerular filtration rate; HF = heart failure; LBBB = left bundle branch block; LVEF = left ventricle ejection fraction; LVRR = left ventricular reverse remodeling; NTproBNP = N-terminal prohormone of brain natriuretic peptide.
