# Supplementary material for: Clinical Determinants and Prognosis of Left Ventricular Reverse Remodelling in Non-Ischemic Dilated Cardiomyopathy
Source: J Cardiovasc Dev Dis. 2022 Jan 11;9(1):20. doi: 10.3390/jcdd9010020 (PMC8778173; doi:10.3390/jcdd9010020)
Supplement: Supplementary file 1 [file jcdd-09-00020-s001.zip › jcdd-1476998-supplementary/Supplemental TableS5.pdf]

Table S5. Analysis of potential predictors of LVRR in patients with the follow-up echocardiogram performed 1 year or less after the initial echocardiogram (N=180).

**Variable**	**Multivariate analysis, NTproBNP included**			**Multivariate analysis, NTproBNP not included**		
	**OR**	**95% CI**	**P value**	**OR**	**95% CI**	**P value**
Age				0.96	0.93-0.99	0.019
Hypertension				2.23	1.05-4.77	0.037
logNT-proBNP (ng/L)	0.72	0.49-1.06	0.100			
logHF duration (months)	0.58	0.44-0.76	<0.001	0.61	0.58-0.76	<0.001
Initial LVEF (%)	0.84	0.77-0.92	<0.001	0.89	0.84-0.95	<0.001
QRS complex (ms)				0.98	0.97-0.99	0.043
Absence of LBBB	4.38	1.53-12.52	0.006			

Data presented as odds ratios and 95% confidence intervals from the logistic regression models. Abbreviations: HF = heart failure; iLVEDD = indexed left ventricular end-diastolic diameter; LBBB = left bundle branch block; LVEF = left ventricle ejection fraction; LVRR = left ventricular reverse remodeling; NTproBNP = N-terminal prohormone of brain natriuretic peptide.
