# Supplementary material for: Clinical Determinants and Prognosis of Left Ventricular Reverse Remodelling in Non-Ischemic Dilated Cardiomyopathy
Source: J Cardiovasc Dev Dis. 2022 Jan 11;9(1):20. doi: 10.3390/jcdd9010020 (PMC8778173; doi:10.3390/jcdd9010020)
Supplement: Supplementary file 1 [file jcdd-09-00020-s001.zip › jcdd-1476998-supplementary/Supplemental TableS6.pdf]

Table S6. Analysis of potential predictors of LVRR in patients with the follow-up echocardiogram performed 1 year after the initial echocardiogram (N=347).

**Variable**	**Multivariate analysis, NTproBNP included**			**Multivariate analysis, NTproBNP not included**		
	**OR**	**95% CI**	**P value**	**OR**	**95% CI**	**P value**
Hypertension				1.55	0.95-2.54	0.077
eGFR (ml/min)	0.98	0.97-0.99	0.012			
logNT-proBNP (ng/L)	0.62	0.47-0.81	<0.001			
logHF duration (months)	0.83	0.69-0.98	0.031	0.81	0.71-0.93	0.002
Initial iLVEDD (mm)				0.95	0.90-0.99	0.032
Initial LVEF (%)	0.93	0.89-0.97	0.001	0.93	0.89-0.96	<0.001
Absence of LBBB	2.96	1.37-6.37	0.006	3.04	1.57-5.87	0.001

Data presented as odds ratios and 95% confidence intervals from the logistic regression models. Abbreviations: eGFR = estimated glomerular filtration rate; HF = heart failure; iLVEDD = indexed left ventricular end-diastolic diameter; LBBB = left bundle branch block; LVEF = left ventricle ejection fraction; LVRR = left ventricular reverse remodeling; NTproBNP = N-terminal prohormone of brain natriuretic peptide.
